# Treatment of Hemorrhoids

**Published:** 1852-02

**Authors:** 


*Treatment of Hemorrhoids.*—In the October number of the Stethoscope, Dr. Thweatt records the result of a trial made with nitric acid (officinal preparation) in this disease. The patient had been afflicted with bleeding piles during fifteen years, with a prolapsus recti on each attempt at stool. He had undergone every plan of treatment. Dr. Thweatt recommended cauterization with nitric acid, to which he assented. It was done by penciling the tumors until it produced a change of color. The operation produced very little pain, and the parts were dressed with lint and sweet oil. On the second day he was entirely free from pain; the piles were less congested and slightly diminished in size; the bright red color was changed to a dirty brown. He was made to strain at stool, which act brought into view tumors situated higher in the intestines. These were cauterized as the first, a straining, burning pain being excited. On the third day the piles were again touched. A short time after the last penciling he was well; no tumors, either internal or external, and there was no further prolapse of the gut.—*New York Medical Gazette.*

